# The Pharmacotherapy of Alcohol Dependence: A State of the Art Review

**DOI:** 10.4103/0973-1229.58820

**Published:** 2010

**Authors:** Avinash De Sousa

**Affiliations:** * *Consultant Psychiatrist and Psychotherapist, Mumbai*

**Keywords:** *Alcohol Dependence*, *Psychopharmacology*, *Naltrexone*, *Disulfiram*, *Topiramate*, *Acamprosate*, *SSRIs*

## Abstract

The psychopharmacology of alcohol dependence is today poised at interesting crossroads. Three major drugs Naltrexone, Disulfiram and Acamprosate have been tried and tested in various trials and have many meta-analyses each to support them. While Naltrexone may reduce craving, Acamprosate scores on cost effectiveness worldwide with Disulfiram being an alcohol deterrent drug. Studies support, refute and criticize the use of each of these drugs. Combining one or more of them is also a trend seen. The most important factor in efficacy has been the combination of psychosocial treatment with medication. Studies from the early 1970s to date have been reviewed and the findings presented in a manner useful for the busy clinician to judge the best pharmacological option in the management of alcohol dependence. The role of depot disulfiram, naltrexone, and medications like Topiramate and SSRIs under research for alcohol dependence, are also addressed.

## Introduction

Alcohol dependence is a major mental health problem in India. About five to seven per cent of the Indian population has been estimated to abuse alcohol and 10-20 million people estimated to be in need of treatment for alcohol dependence, with alcohol dependence accounting for 1.2% of the total deaths in India (Grover, Bhateja and Basu, 2007). This is a little lower than the three to five per cent estimate for major world populations (Murray and Lopez, 1997; Makela, Martikainen and Nihtila, 2005). It has also been seen that only 8-10% of total alcohol dependence patients are treated in specialty settings and less than 10% receive pharmacotherapy (Finney, Hahn and Moos, 1996). Today, with a better understanding of the basic neurobiological components of alcohol dependence, we have pharmacological agents targeted at improving drinking behavior, enhancing abstinence and preventing relapse as well as reducing the amount of alcohol people drink when they relapse (Swift, 1999; Rosenthal, 2006).

There are currently three US FDA approved medications for the relapse prevention of alcohol dependence. These are Disulfiram, Naltrexone and Acamprosate. This paper shall review the clinical experiences with these drugs, discuss the more recent trends in combination therapies and also briefly describe some new promising experimental pharmacological therapies for the treatment of alcohol dependence.

## Naltrexone

Naltrexone is an opioid receptor antagonist approved by the US FDA in 1994 that reduces heavy drinking by diminishing the rewarding neurobiological effects of alcohol (Harris and Erickson, 1979; Gianoulakis, 1993; Pettinati, *et al*., 2006; Anton, 2008). It acts via reducing dopamine release in response to alcohol from the dopamine reward pathways in the ventral tegmental area and the nucleus accumbens (Gessa, *et al*., 1985; Heimer and Alheid, 1991; Benjamin, *et al*., 1993). It is also known to reduce the release of endogenous opioids like endorphins in response to alcohol (Herz, 1997).

Naltrexone is known to reduce craving for alcohol in both alcohol dependent patients (Monti, *et al*., 1999) and social drinkers (Davidson, *et al*., 1996; Richardson, *et al*., 2008). There are also a number of reviews and meta-analyses that support naltrexone as a treatment for alcohol dependence, along with 29 published randomized placebo controlled trials, some supportive and some not (Garbutt, *et al*., 1999; Melchior and Hoes, 1999; Kranzler, 2000; Streeton and Wheelan, 2001; Richardson, *et al*., 2008). Two pivotal early trials were the first to illustrate the efficacy of naltrexone in the management of alcohol dependence (Volpicelli, *et al*., 1992; O’Malley, *et al*., 1992). A recent review has reported modest favorable effects of naltrexone on heavy drinkers. In a majority of the studies reviewed, Naltrexone reduces significantly the rates of drinking in heavy drinkers by 30-60%. It reduces craving significantly though abstinence is seen usually in 25-35% cases. (Pettinati, *et al*., 2006). Another meta-analysis, a Cochrane collaborative, found a decrease in the rate of relapse in alcohol dependence in case of short-term studies (< 12 weeks), but the effect on increasing abstinence was small. Medium term trials (> 12 weeks) are too limited in number (eight studies) to show benefits for relapse prevention but do show increased time to the consumption of the first drink and decreased craving over time (Srisurapanont and Jarasuraisin, 2005). The dosage used in most studies is 50-100mg per day. More often than not, naltrexone was used in combination with cognitive behavioral therapy, supportive individual or group psychotherapy and relapse prevention therapy. Naltrexone alone has been shown to reduce heavy drinking rates in a smoking cessation program (King, *et al*., 2009) while it has also been shown to improve the cost effectiveness of cognitive behavioral therapy in alcohol dependence (Walters *et al*., 2009).

Naltrexone also has a favorable safety profile. It does not reduce seizure threshold nor have any fatalities been reported with naltrexone overdose. It has not been associated with pleasurable effects, does not result in tolerance and has no abuse potential (Swift, *et al*., 1994; Croop, Faulkner and Labriola, 1997; no recent citable studies done in the author’s knowledge). The frequency of side effects with naltrexone is low, with nausea and vomiting being the most commonly reported, followed by headache, low energy, decreased alertness, depression and anxiety. These side effects resolve in one to two days after starting naltrexone, or after a few doses, or on reducing the daily dosage (Croop, Faulkner and Labriola, 1997). Though naltrexone carries a black box warning of possible hepatotoxicity, there are no reports of hepatotoxicity with the recommended daily dosages (Yen, *et al*., 2006). In fact, liver enzymes which are raised often reduce with naltrexone due to decreased alcohol consumption (Berg, *et al*., 1996; Yen, *et al*., 2006).

From the clinical perspective, it is essential to note that though naltrexone has been used widely in the management of alcohol dependence there are always potential barriers to naltrexone response. Among these, the most common are medication non-adherence and heterogeneity of the alcohol dependent patient population. There are also probably different endophenotypes of alcoholism whereby some patients would respond differentially to naltrexone. We are already aware of naltrexone responders associated with response to alcohol in the laboratory (King, *et al*., 1997), family history of alcoholism (Jaffe, *et al*., 1996) and genotypes (Oslin, *et al*., 2003).

A long acting extended release injectable formulation of Naltrexone (encapsulated naltrexone 380mg in bio-degradable microspheres) was approved by the European FDA in 1996 for the treatment of alcohol dependence. The preparation was shown to maintain therapeutic levels for a month after injection. It also had reduced side effects and less chance of hepatic toxicity as it eliminated first pass metabolism in the liver (Garbutt, *et al*., 2005). Most studies with the preparation in keeping with oral naltrexone find reduction in the time to relapse in alcohol dependent patients (Johnson, *et al*., 2004; Kranzler, *et al*., 2004).

## Acamprosate

Acamprosate (calcium acetylhomotaurinate) is a synthetic compound with a chemical structure similar to the amino acid neurotransmitter GABA and amino acid neuromodulator taurine. It is available as 333mg tablets with recommended dosages being two 333mg tablets thrice a day. It has been used to treat over 1.5 million patients since its introduction in 1989 and is currently available and prescribed in over 28 countries (Mason, 2001). It is hypothesized that acamprosate acts via modulation of glutamatergic hyperactivity associated with chronic alcohol induced changes (De Witte *et al*., 2005). Acute alcohol intake disrupts the normal balance between neuronal excitation and inhibition regulated by GABA, glutamate and other receptor systems. This results in an exaggeration of the inhibitory processes. During chronic alcohol consumption, neuroadaptation occurs via up regulation of excitatory NMDA receptors. Abrupt removal of alcohol leaves the up regulated NMDA system unopposed in a state of hyperactivity. Acamprosate may act at the regulatory sites on both ionotropic and metabotropic NMDA receptors and normalizes this hyper excitability to re-establish homeostasis (Hoffman, 2003; De Witte, 2004).

The US FDA approval of acamprosate was based on three trials that showed acamprosate’s efficacy in reducing relapse and maintaining abstinence in patients with alcohol dependence (Paille, *et al*., 1995; Sass, *et al*., 1996; Pele, *et al*., 1997). A number of reviews and meta-analyses have demonstrated moderate efficacy of acamprosate in the management of patients with alcohol dependence (Bouza, *et al*., 2004; Mason, 2005). The clinical efficacy of acamprosate was evaluated in a systematic review of published clinical trials up to 1997 with a consistent finding being 30-50% increase in non drinking days (Wilde and Wagstaff, 1997). A relatively recent meta analysis of 22 studies also reported the same effect (Mann *et al*., 2004). In summary, there is good evidence to support increased abstinence and decreased drinking days with acamprosate compared to placebo in the treatment of alcohol-dependent patients. The strongest effect of acamprosate is seen in recently detoxified alcohol dependents with very good data supporting its efficacy in long term studies.

In addition, all clinical trials support a favorable safety and tolerability profile of acamprosate. It has no abuse potential and the only side effect noted in all studies and on an overdose is diarrhea while hypercalcemia may be seen in chronic overdose cases only (Mason, 1996; Mann, 2004). Studies have also shown acamprosate to be a cost effective treatment (Schadlich and Brecht, 1998). However, in India, Disulfiram is cheaper than either Naltrexone or Acamprosate. Hence Disulfiram is the more cost effective treatment. Worldwide, though, Naltrexone and Acamprosate are cheaper than Disulfiram, and Disulfiram is not easily available in many nations.

## Combined Naltrexone and Acamprosate Therapy

Some authors have suggested a clinical rationale for combining Naltrexone and Acamprosate therapy in the management of alcohol dependence as they act on different neurotransmitter systems (Mason, 2005; Kranzler, 2006). Clinical trials have proved that the above combination is better than acamprosate alone, though not better than naltrexone alone, especially in terms of first relapse. These were also the first studies to suggest that combined therapy is better than monotherapy in the treatment of alcohol dependence (Kiefer *et al*., 2003; Feeney *et al*., 2006). More recent researches may be underway but have not been published at this point of time in the author’s knowledge.

## Disulfiram

Disulfiram, an aldehyde dehydrogenase inhibitor, has been approved by US FDA in 1951 as an aversive therapy for the management of alcohol dependence. It blocks the oxidation of ingested alcohol at the acetaldehyde stage and prevents its rapid metabolism to acetate. Thus when a disulfiram treated patient ingests even small amounts of alcohol, acetaldehyde accumulates as a result of the disulfiram-ethanol reaction and causes tachycardia, hypotension, diaphoresis, flushing, dyspnea, nausea and vomiting. These symptoms act as a deterrent to alcohol ingestion (Savas and Gullu, 1997). Disulfiram is available as 250mg tablets with the recommended dosage being 250-500mg per day. Disulfiram is often not recommended as the first line medication for newly diagnosed alcohol dependent patients but is reserved for treating patients who have previously failed one or more courses of treatment or those who are motivated to achieve complete abstinence (Fuller, *et al*., 1986). With the advent and emergence of Naltrexone and Acamprosate, there has been a decline in Disulfiram use with it slipping to a second line treatment in many centers for the treatment of alcohol dependence. Safety concerns may also be the reason for this as many alcoholic patients try to consume alcohol even when on Disulfiram and hence may cause themselves unnecessary harm.

Several reviews support the efficacy of supervised use of Disulfiram in the management of alcohol dependence (Banys, 1988; Brewer, 1995; Suh *et al*., 2006). This background alone ought to make everyone in the world of alcoholism treatment aware of disulfiram’s potential especially for the large and often demoralizing number of patients that do not respond to other treatments. There is no doubt that supervised disulfiram therapy is an integral component of any alcohol treatment program (Chick and Brewer, 1999). There are also a large number of limitations with respect to disulfiram research. In a review of studies published between 1948 and 1971 it was seen that among 42 studies only one had an adequate research design. The limitations still hold, with no randomized double blind trials being ever conducted on Disulfiram in this reviewer’s knowledge. Lack of double blinded controlled and randomized clinical trials with disulfiram is another hindrance. The reason for this is that awareness that the patient is on disulfiram is an essential for the action of disulfiram in order to enhance its efficacy and hence most studies are open ones (Lundwall and Baekeland, 1971; Hughes and Cook, 1997; Fuller and Gordis, 2004). It has been noted that disulfiram is most effective when supervised and used in alcoholism with the help of a close family member (Brewer, 2005).

Disulfiram is an old drug, long out of patent protection. It is thus cheap and marketed by manufacturers of the generic drug who do little advertising and research. There is also a ready availability of funding for naltrexone and acamprosate research which means that researchers with projects on these drugs are more likely to study acamprosate or naltrexone than disulfiram. Fears of disulfiram hepatotoxicity are often exaggerated. There is about one case in 25000 patient years (Poulsen, 1992; Brewer and Hardt, 1999; Martin and Beresford, 2007). Death from disulfiram is also very rare (Brewer, 2005). Disulfiram can be readily prescribed with Acamprosate and Naltrexone and is shown to improve the efficacy of Acamprosate (Beeson *et al*., 1998; Suh *et al*., 2006). There have been cases where disulfiram has been continued safely for over 15 years (Brewer, 1993; Garbutt, 2009).

A long acting depot preparation of Disulfiram in the form of a Disulfiram implant is also available. It was introduced in the 1950s and is still used in some parts of the world. In various studies, it shows a similar pharmacological and clinical profile like oral Disulfiram (Johnsen and Marland, 1991; 1992). No current research evidence is available on this preparation.

## Comparison of the Three Drugs

There are only a few studies that provide a head on between the above three drugs. Naltrexone has been proven superior to Acamprosate in one of the earliest studies comparing the two drugs (Rubio *et al*., 2001). An Indian study has shown Naltrexone to be superior to Disulfiram (Naidu *et al*., 2000); while a retrospective chart review has shown acamprosate to be superior to naltrexone (Basu *et al*., 2005). No major recent studies add to this literature and future research in this area is warranted.

The author of this article has been involved in recent work on Disulfiram. In similar studies the author and others have shown disulfiram to be superior to naltrexone, acamprosate and topiramate in separate studies. They, along with others, have also shown disulfiram to be superior to naltrexone in the management of adolescents with alcohol dependence. (De Sousa and De Sousa, 2004; De Sousa and De Sousa, 2005; Petrakis *et al*., 2006; Pettinati *et al*., 2008; Laaksonen *et al*., 2008; De Sousa, De Sousa and Kapoor, 2008; De Sousa and De Sousa, 2008). Further studies on similar lines are already in progress.

## Topiramate

Though not US FDA-approved, there are various reports of topiramate being effective in the management of alcohol dependence. It is a drug that has been used successfully in epilepsy and migraine. It is postulated to be effective in the management of alcohol dependence as it reduces dopamine release after alcohol consumption due to its ability to enhance GABA mediated inhibition through non benzodiazepine receptors (White *et al*., 2000; Johnson *et al*., 2003). There is also a possible glutamate antagonism mechanism at the alpha amino 3 hydroxy 5 methyl 4 isoxazole propionic acid receptors (Skradiski and White, 2000). Various randomized double blind controlled trials have reported the efficacy of topiramate in reducing the percentage of drinking days and in maintaining abstinence (Johnson *et al*., 2003; Rosenthal, 2006; Garbutt, 2006). Topiramate has been used in doses of 100-250mg per day. The drug in most studies has been used alone with conflicting results. While some studies suggest a moderate efficacy others regard it as ineffective. No specific indications, contra-indications or specific sub groups of alcohol dependent patients that may respond to Topiramate have yet been delineated.

## Future Probable Pharmacological Agents

Several other classes of drugs, not yet currently approved, are under active study for use in alcohol dependence. Selective serotonin reuptake inhibitors (SSRIs) that augment brain serotenergic function have shown to reduce alcohol consumption in animal studies. Findings with alcohol dependent patients are inconsistent but these drugs are the main stay in the management of depression with alcohol dependence. Type A alcoholics (late onset, less severe dependence, less psychopathology) show a favorable response to these drugs. The two drugs that have been studied so far are Sertraline and Fluoxetine in one study each (Kranzler *et al*., 1996; Anton and Swift, 2003; Nunes and Levin, 2004; Dundon *et al*., 2004). More recent work has not been reported.

A few studies have also studied the role of 5HT-3 receptor antagonist Ondansetron with very modest results in the management of alcohol dependence (Johnson, *et al*., 2000; no more recent work available).

Acute alcohol consumption increases dopamine release from the nucleus accumbens while chronic alcohol consumption decreases mesostriatal dopamine activity in rodents and decreases dopamine and its metabolites in alcoholics (DiChiara and Imperato, 1985; Fulton *et al*., 1995). Medications that affect dopamine may thus have a potential role in alcohol dependence. Some studies have shown that the dopamine receptor antagonist, haloperidol, reduces the stimulating and euphorigenic effects of alcohol in social drinkers and reduces craving in pretreated alcoholics (Modell *et al*., 1993). Studies have shown mild to modest results in a similar manner with the use of antipsychotics like Clozapine, Olanzapine, Fluphenthixol and Amisulpride in separate settings (Drake *et al*., 2000; Wisebeck *et al*., 2001; Marra *et al*., 2002; Guardia *et al*., 2004).

Pharmacotherapy for alcohol dependence is always delivered in a psychosocial context that may affect the outcome of the treatment. The rigorous study of different psychotherapeutic treatments for alcohol dependence has shown several distinct approaches to be effective. Many psychosocial interventions for alcohol dependence, including Alcoholics Anonymous, can be integrated successfully with pharmacotherapy. Psychosocial interventions, ranging from brief medical management to more intensive individualized psychotherapies, have all been shown to produce positive outcomes in certain studies, depending on the specific medication and the study context. Particularly successful combinations may include the use of behavioral marital therapy plus a disulfiram contract for patients taking that medication, and the combination of naltrexone or acamprosate with cognitive-behavioral therapy or psychosocial support. Ongoing research examining the optimal combinations of medications with different psychosocial treatments for alcohol dependence may further inform the field (Weiss and Kueppenbender, 2006).

**Figure 1:**
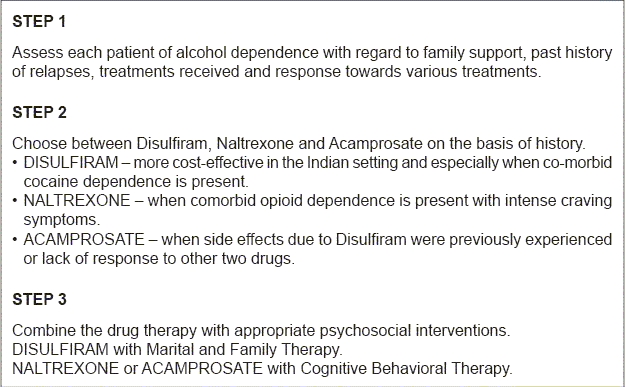
Flowchart of the paper

## Concluding Remarks

Today the pharmacology scenario with respect to alcohol dependence is more promising than ever. The molecules used regularly in the management of alcohol dependence have been widely researched such that the sharp clinician can ascertain their use for specific patient populations when needed. Further studies involving comparisons of various different drugs across various settings and subsets of the alcoholic population are needed to clear any lacunae that remain.

### Thumb rule for clinicians

It is important for clinicians treating alcohol dependence to note the following:

- Naltrexone and Acamprosate, though effective, only reduce craving and do not deter the patient from taking alcohol. He may still drink while on these drugs with no untoward effects.
- Disulfiram, though underused, is cheaper than the above two drugs but very effective as an alcohol deterrent as fear of a disulfiram ethanol reaction forces the patient to be off alcohol when on the drug.
- Combination therapy of disulfiram with any of the other drugs thereby acting on different neurobiological systems may be optimal for the effective management of alcohol dependence.
- The use of psycho-education about disulfiram and its actions is very essential to get the best effects out of the medication.

### Take home message

The pharmacotherapy of alcohol dependence is keenly poised today. We have different drugs (Naltrexone, Disulfiram and Acamprosate) with varying mechanisms of action aimed at different populations which, when used judiciously, can bring about good results along with psychosocial interventions in the management of alcohol dependence. Newer agents like Topiramate and SSRIs are being investigated for their role in alcohol dependence.

### Conflict of interest

None declared.

No funding, speaker fees or research grant received.

### Declaration

This article is my original unpublished work and has not been sent for publication to any other journal.

## Questions That This Paper Raises

1. Which is the best drug for the long-term management of alcohol dependence?
2. Is one drug enough or do we need multiple drug therapy in the long-term management of alcohol dependence?
3. Does Disulfiram have a role in the modern day pharmacotherapy of alcohol dependence?
4. Do newer drugs other than those approved by US FDA play a role in the management of alcohol dependence?
5. Do the findings from studies worldwide hold good when considering Indian patients with alcohol dependence?

## About the Author

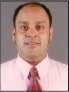



Dr. Avinash De Sousa is a consultant psychiatrist and psychotherapist with a private practice in Mumbai. He is an avid reader and has over 50 publications in national and international journals. His main areas of interest are alcohol dependence, child and adolescent psychiatry, mental retardation, autism and developmental disabilities. He is also the academic director of the Institute of Psychotherapy Training and Management, Mumbai. He actively teaches psychiatry, child psychology and psychotherapy at over 18 institutions as a visiting faculty.
